# Clinical Usefulness of Procalcitonin and C-Reactive Protein as Outcome Predictors in Critically Ill Patients with Severe Sepsis and Septic Shock

**DOI:** 10.1371/journal.pone.0138150

**Published:** 2015-09-14

**Authors:** Jeong-Am Ryu, Jeong Hoon Yang, Daesang Lee, Chi-Min Park, Gee Young Suh, Kyeongman Jeon, Joongbum Cho, Sun Young Baek, Keumhee C. Carriere, Chi Ryang Chung

**Affiliations:** 1 Department of Critical Care Medicine, Samsung Medical Center, Sungkyunkwan University School of Medicine, Seoul, Republic of Korea; 2 Division of Cardiology, Department of Medicine, Samsung Medical Center, Sungkyunkwan University School of Medicine, Seoul, Republic of Korea; 3 Department of Surgery, Samsung Medical Center, Sungkyunkwan University School of Medicine, Seoul, Republic of Korea; 4 Division of Pulmonary and Critical Care Medicine, Department of Medicine, Samsung Medical Center, Sungkyunkwan University School of Medicine, Seoul, Korea; 5 Biostatistics and Clinical Epidemiology Center, Samsung Medical Center, Seoul, Korea; 6 Department of Mathematical and Statistical Sciences, University of Alberta, Edmonton, Canada; Universidade do Extremo Sul Catarinense, BRAZIL

## Abstract

Sepsis is a major cause of mortality and morbidity in critically ill patients. Procalcitonin (PCT) and C-reactive protein (CRP) are the most frequently used biomarkers in sepsis. We investigated changes in PCT and CRP concentrations in critically ill patients with sepsis to determine which biochemical marker better predicts outcome. We retrospectively analyzed 171 episodes in 157 patients with severe sepsis and septic shock who were admitted to the Samsung Medical Center intensive care unit from March 2013 to February 2014. The primary endpoint was patient outcome within 7 days from ICU admission (treatment failure). The secondary endpoint was 28-day mortality. Severe sepsis was observed in 42 (25%) episodes from 41 patients, and septic shock was observed in 129 (75%) episodes from 120 patients. Fifty-five (32%) episodes from 42 patients had clinically-documented infection, and 116 (68%) episodes from 99 patients had microbiologically-documented infection. Initial peak PCT and CRP levels were not associated with treatment failure and 28-day mortality. However, PCT clearance (PCTc) and CRP (CRPc) clearance were significantly associated with treatment failure (*p* = 0.027 and *p* = 0.030, respectively) and marginally significant with 28-day mortality (*p* = 0.064 and *p* = 0.062, respectively). The AUC for prediction of treatment success was 0.71 (95% CI, 0.61–0.82) for PCTc and 0.71 (95% CI, 0.61–0.81) for CRPc. The AUC for survival prediction was 0.77 (95% CI, 0.66–0.88) for PCTc and 0.77 (95% CI, 0.67–0.88) for CRPc. Changes in PCT and CRP concentrations were associated with outcomes of critically ill septic patients. CRP may not be inferior to PCT in predicting outcome in these patients.

## Introduction

Sepsis is a major cause of mortality and morbidity in critically ill patients. Procalcitonin (PCT) and C-reactive protein (CRP) are the most frequently used biomarkers for critically ill patients with sepsis [1, 2]. PCT is considered to have a higher capacity to diagnose sepsis than CRP [1–5]. CRP has been suggested to predict therapeutic response and outcome in sepsis, and slower changes have been associated with persistent infection, organ failure, or mortality in the intensive care unit (ICU) [6]. A relatively rapid fall in PCT may be associated with beneficial outcomes of pneumonia, meningitis, and burn-associated, or other infections. Elevated PCT concentrations may be associated with organ failure and mortality and thus might have predictive value [6].

Antimicrobial therapy that is promptly administered and controlling the source of infection have been shown to improve outcome in septic patients [7]. If sepsis is well controlled, PCT and CRP may show decreasing patterns. Therefore, changes in biochemical markers may be useful in predicting therapeutic response and prognosis in septic patients. However, the predictive value of these markers is not yet clear [8]. Some studies have shown that it is possible to predict the outcome of sepsis based on changes in PCT and CRP [1, 2, 4, 6, 9]. A dynamic approach of assessing these biomarkers may provide more information on the outcome of patients with sepsis. A recent study suggested that procalcitonin clearance (PCTc) could be used to monitor the evolution of PCT levels during severe sepsis [10].

When septic patients are admitted to the ICU, these biochemical markers are frequently evaluated to predict treatment response, infection severity, and patient outcome. However, the prognostic values of these biochemical markers are still debated. We investigated predictive values based on changes in PCT and CRP concentrations in critically ill patients with severe sepsis/septic shock to determine which biochemical marker better predicts outcome.

## Methods

### Ethics statement

This retrospective study was performed in a cohort of patients with sepsis who were admitted to the medical or oncology ICUs of Samsung Medical Center (a 1961-bed, university-affiliated, tertiary referral hospital in Seoul, South Korea) from March 2013 to February 2014. The study was approved by the Institutional Review Board of Samsung Medical Center (SMC 2014-06-130-001) according to the Declaration of Helsinki to review and publish information from the patients' records. Informed consent was waived because of the retrospective nature of the study. The patients’ records/information was anonymized and de-identified prior to analysis.

### Patients

We retrospectively analyzed 171 episodes in 157 patients with sepsis. Adult patients who were older than 18 years and fulfilled the definitions of severe sepsis or septic shock were recruited. Patients were excluded if they had a history of trauma, surgery, or a ‘do not resuscitate’ (DNR) order on ICU admission. All patients received standard supportive treatment following the recommendations of the Surviving Sepsis Campaign released in 2012 [11].

### Endpoints and definitions

The primary endpoint of this study was patient outcome within 7 days from ICU admission (treatment failure). Treatment success without modification was defined as symptoms that disappeared or were cured by the initial treatment within 7 days from ICU admission. Treatment success with modification was defined as elimination of the episode’s fever or signs of infection upon the addition of other anti-infective drugs or treatments within 7 days. Treatment failure was defined as fever that persisted for 7 days or reappeared, bacteremia that persisted or reappeared, or progression of infection, as evidenced by worsening of the source of infection, the appearance of signs or symptoms of septic shock, or death [12, 13]. The secondary endpoint of this study was 28-day mortality. ICU-free days were defined as the number of days between successful transfer to a normal ward or 28 days after study enrollment. Therefore, the number of ICU-free days was 0 if the patient died before day 28 or stayed in the ICU for 28 days or longer [14].

Initial blood samples were collected within 12 hours of ICU admission. The day of ICU admission was defined as day 0. We collected laboratory data on days -1 to day 7. Serum PCT concentrations were measured using enzyme-linked fluorescent assays (Brahms Diagnostica GmbH, Berlin, Germany), and the lower reference limit was 0.05 ng/mL. Serum CRP concentrations were measured using immunoturbidimetric assays (CRPL3, Roche Diagnostics, Indianapolis, IN, USA), and the lower reference limit was 0.3 mg/dL. We defined initial baseline PCT and CRP levels as peak levels from days -1 to 2 and subsequent levels as minimal levels from days 5 to 7. PCT and CRP kinetics are expressed as ΔPCT and ΔCRP concentrations, which are the differences between baseline and subsequent measurements. ΔPCT and ΔCRP are calculated as baseline levels minus subsequent levels. PCTc was calculated as the percentage of ΔPCT over baseline PCT level. CRP clearance (CRPc) was calculated in the same way.

Sepsis, severe sepsis, and septic shock were defined according to the American College of Chest Physician/Society of Critical Care Medicine (ACCP/SCCM) guidelines [15, 16]. Fever is defined as a single oral temperature measurement > 38.3°C (101°F) or a temperature of > 38.0°C (100.4°F) sustained over a one-hour period. Neutropenia is defined as an absolute neutrophil count (ANC) of < 500 cells/mm^3^ or an ANC that is expected to decrease to < 500 cells/mm^3^ during the next 48 hours [17]. Bacteremia was diagnosed when one positive culture was obtained, except for coagulase-negative Staphylococci, for which at least two positive blood cultures were required [12]. Clinically-documented infection (CDI) was defined as a febrile episode with a focal infection that was not accessible to sampling or was sampled with negative microbiological results, and/or radiological findings suggestive of infection. Microbiologically-documented infection (MDI) was defined as a febrile episode with a positive microbiological assessment (causative pathogen) from a focus of infection and/or blood culture [12, 13]. On ICU admission, illness severity was assessed using the simplified acute physiology score 3 (SAPS 3) and the severity of multiple organ dysfunction syndrome was evaluated using sequential organ failure assessment (SOFA) scores from the worst data point of the first 24 hours in the ICU.

Patients with malignancies were included in this study; all definitions associated with cancer status used previously reported definitions [18–21]. Patients with relapsed malignancies following intensive front-line chemotherapy or who failed to respond to initial chemotherapy were considered as relapsed/refractory status. The extent of malignancy was classified according to tumor extent and major organ involvement as reported previously [18–20]. Extensive disease was defined as stage III or IV for lymphoma, metastatic or locally extensive disease for solid malignancies, and greater than 80% blasts in bone marrow, greater than 25,000 blasts/μL in peripheral blood, or the need for leukapheresis for hematologic malignancies. Major organ involvement was defined as a pathologically confirmed or radiologically suspected invasion of the brain, heart, lung, liver, or kidney [19, 22]. Recent chemotherapy and recent radiation were defined as treatment within the past four weeks [23].

### Statistical analyses

Although multiple episodes are possible per each patient, we treated each episode as an independent observation, because there were only 12 of 157 (8%) patients with repeated episodes. Variables are expressed as numbers and percentages, or medians and interquartile ranges (IQRs). The predictive performance of each biochemical marker was assessed using the area under the curve (AUC) of receiver operating characteristic (ROC) curves of the sensitivity over 1-specificity. AUCs were compared using the nonparametric approach of DeLong et al. [24] for two correlated AUCs. Kaplan-Meier method was used to determine the survival curves, which were then compared using log-rank tests for survival data. Both univariate and multiple logistic regression analysis methods were used to predict the success of a treatment and the 28-day mortality. Cox proportional hazard model was used to analyze the effects of covariates on survival times. Model goodness of fit was assessed using the Hosmer-Lemeshow test and competing models were compared using a Chi-square test. Data were analyzed using Statistical Analysis System (SAS) version 9.4 (SAS Institute, Cary, NC)

## Results

### Patient characteristics

A total of 171 episodes in 157 patients were analyzed. The median age of patients was 62 years (range, 54~71), and 112 (66%) episodes came from 102 male patients. Of these, 126 (73%) episodes from 112 patients had malignancies, among which 57 (33%) episodes from 54 patients had solid tumors and 69 (40%) episodes from 58 patients had hematologic malignancies. Among episodes with solid tumors, 20 (18 patients) had lung cancer, eight (7 patients) had hepatic cancer, seven (7 patients) had gastric cancer, four (4 patients) had colorectal cancer, and three (3 patients) had breast cancer. Fifteen episodes (15 patients) had other types. Among episodes with hematologic malignancies, 29 (26 patients) had leukemia, 30 (22 patients) had lymphoma, two (2 patients) had myelodysplastic syndrome, and eight (8 patients) had multiple myeloma. Of the episodes with malignancies, 29 (17%) episodes from 27 patients were newly diagnosed and 71 (42%) from 64 patients were in a relapsed/refractory state. Extensive disease was noted in 73 episodes (43%) from 64 patients. Major organ involvement was noted in 30 (18%) episodes from 27 patients, including the brain [11 episodes (6%)], lung [6 episodes (4%)], liver [9 episodes (5%)], and kidney [1 episode (1%)]. Three episodes (2%) had multiple organ involvement. The baseline characteristics of episode are shown in Table 1.

**Table 1:** Baseline characteristics on ICU admission.

Variables			No. of episodes (%)
Age (years)			62 (54–71)*
Gender (male)			112 (66)
Underlying diseases			
	Diabetes		54 (32)
	Hypertension		54 (32)
	Cirrhosis		18 (11)
	Renal failure		9 (5)
	Malignancy		126 (73)
	Ischemic heart disease		3 (2)
	Heart failure		4 (2)
	Stroke		8 (5)
	COPD		6 (4)
	Interstitial lung disease		3 (2)
	Old tuberculosis		14 (8)
	Other		22 (13)
Type of malignancy			
	Solid		57 (33)
	Hematologic		69 (40)
Malignancy status			
	First presentation		29 (17)
	Relapsed/refractory		71 (42)
	Extensive disease		73 (43)
	Major organ involvement		30 (18)
	Stem cell transplantation		10 (6)
		Allogenic	4 (2)
		Autologous	6 (4)
	Recent chemotherapy		79 (46)
	Recent radiation therapy		2 (1)
	Duration of malignancy (months)		202 (44–632)*
Illness severity			
	SAPS 3		78 (67–87)*
	SOFA score		11 (8–14)*
Need for mechanical ventilator			91 (53)
Need for renal replacement therapy			41 (24)
Need for vasopressor support			131 (77)

COPD, chronic obstructive pulmonary disease; SAPS 3, simplified acute physiology score 3; SOFA, sequential organ failure assessment.

*Continuous variables are summarized as a median and the interquartile range.

Severe sepsis was verified in 42 (25%) episodes from 41 patients and septic shock was observed in 129 (75%) episodes from 120 patients. Moreover, 55 (32%) episodes from 42 patients had clinically-documented infection (CDI) and 116 (68%) episodes from 99 patients had microbiologically-documented infection (MDI). Febrile neutropenia was noted in 55 (32%) episodes from 51 patients. The primary septic origin was lung and abdomen (54%). The episode characteristics are shown in Table 2.

**Table 2:** Episode characteristics.

Variables			No. of episodes (%)
Classification of sepsis severity			
	Severe sepsis		42 (25)
	Septic shock		129 (75)
Classification of sepsis			
	Clinically-documented infection		55 (32)
	Microbiologically-documented infection		116 (68)
Febrile neutropenia			55 (32)
Primary origin of sepsis			
	Lung		74 (43)
	Abdomen		19 (11)
	Urinary		10 (6)
	Soft tissue		2 (1)
	Catheter-related bloodstream infection		8 (5)
	Hepatobiliary		12 (7)
	Mixed		15 (9)
	Other		5 (3)
Microbiological documentation			
	Gram-positive bacteria		48 (28)
		Staphylococcus aureus	19 (11)
		Streptococcus pneumoniae	5 (3)
		Staphylococcus spp.	5 (3)
		Streptococcus spp.	3 (2)
		Enterococcus spp.	15 (9)
		Other	1 (1)
	Gram-negative bacteria		92 (54)
		Klebsiella spp.	30 (18)
		Escherichia coli	25 (15)
		Pseudomonas aeruginosa	13 (8)
		Stenotrophomonas maltophilia	9 (5)
		Acinetobacter baumannii	7 (4)
		Enterobacter cloacae	4 (2)
		Other	4 (2)
	Fungi		9 (5)
		Candida spp.	8 (5)
		Cryptococcus neoformans	1 (1)
	Pneumocystis jirovecii		1 (1)
	Tuberculosis		2 (1)
	Combined		29 (17)
	Unknown		55 (32)
Antibiotic therapy			
	Monotherapy		20 (12)
	Combination therapy		151 (88)
Antifungal therapy			38 (22)
Outcomes within 7 days			
	Success without modification		76 (44)
	Success with modification		29 (17)
	Treatment failure		66 (39)
Outcomes			
	28-day mortality		45 (29)
	ICU mortality		37 (24)
	Hospital mortality		58 (37)
	ICU-free days		18.2 (0–23.8)*
	Length of stay in ICU (days)		6.1 (3.2–11.0)*
	Length of stay in hospital (days)		28.8 (14.9–51.1)*

*ICU* intensive care unit;

*Continuous variables are summarized as a median and the interquartile range.

### Outcomes associated with severe sepsis and septic shock

Overall treatment success was observed in 105 (61%) episodes from 98 patients. The 28-day mortality was 29 percent (Table 2). The univariate relationship between each biochemical marker and outcome are shown in Table 3. The initial levels of PCT peak and CRP peak were not associated with outcomes. However, PCTc and CRPc were associated with treatment failure (*p* = 0.007 and *p* < 0.001, respectively) and the 28-day mortality (*p* = 0.005 and *p* = 0.004, respectively).

**Table 3:** Biochemical markers in predicting treatment failure and 28-day mortality.

	Total (n = 171)	Treatment success (n = 105)	Treatment failure (n = 66)	p
PCT Peak -1-2 day	9.9 (2.9–41.1)	14.6 (4.1–60.1)	8.1 (2.3–33.7)	0.066
PCT min 5–7 day	2.1 (0.6–6.8)	2.1 (0.5–7.0)	1.9 (0.7–6.0)	0.693
ΔPCT Peak-min	8.6 (1.7–34.0)	12.8 (2.7–49.6)	5.2 (0.7–28.8)	0.056
PCTc Peak-min	84.1 (63.4–90.9)	87.8 (76.0–91.8)	76.6 (45.8–88.1)	0.007
CRP Peak -1-2 day	19.1 (10.7–28.4)	18.9 (11.3–28.4)	19.2 (9.8–26.0)	0.880
CRP min 5–7 day	4.9 (2.4–10.6)	3.7 (2.1–9.2)	7.6 (3.7–11.4)	0.004
ΔCRP Peak-min	11.7 (5.8–19.1)	13.9 (6.9–20.4)	9.7 (3.2–15.1)	0.005
CRPc Peak-min	67.6 (43.2–85.6)	74.6 (54.3–87.4)	54.2 (24.6–74.8)	< 0.001
	Total (n = 171)	Survivor (n = 125)	Non-survivor (n = 46)	p
PCT Peak -1-2 day	9.9 (2.9–41.1)	10.0 (3.0–54.7)	9.8 (2.3–33.6)	0.134
PCT min 5–7 day	2.1 (0.6–6.8)	2.0 (0.6–7.2)	2.4 (0.6–5.3)	0.615
ΔPCT Peak-min	8.6 (1.7–34.0)	9.5 (2.4–46.9)	5.9 (0.8–27.2)	0.087
PCTc Peak-min	84.1 (63.4–90.9)	87.4 (71.6–92.5)	71.0 (46.5–85.8)	0.005
CRP Peak -1-2 day	19.1 (10.7–28.4)	18.9 (11.1–27.3)	19.3 (9.8–30.6)	0.767
CRP min 5–7 day	4.9 (2.4–10.6)	4.2 (2.2–9.9)	7.6 (3.6–15.0)	0.023
ΔCRP Peak-min	11.7 (5.8–19.1)	13.0 (6.1–19.9)	9.3 (3.0–15.8)	0.056
CRPc Peak-min	67.6 (43.2–85.6)	71.3 (51.5–86.2)	51.0 (25.3–79.5)	0.004

PCT Peak -1-2 day, peak level of procalcitonin from day -1 to day 2; PCT min 5–7 day, minimum level of procalcitonin from day 5 to day 7; ΔPCT Peak-min, peak level of procalcitonin minus minimum level of procalcitonin; PCTc Peak-min, procalcitonin clearance (100 × ΔPCT Peak-min/ PCT Peak -1-2 day); CRP Peak -1-2 day, C-reactive protein level of procalcitonin from day -1 to day 2; CRP min 5–7 day, minimum level of C-reactive protein from day 5 to day 7; ΔCRP Peak-min, peak level of C-reactive protein minus minimum level of C-reactive protein; CRPc Peak-min, C-reactive protein clearance (100 × ΔCRP Peak-min/ CRP Peak -1-2 day).

We also considered multiple logistic and Cox regression models to predict the outcomes of treatment success and 28 mortality by PCTc and CRPc, adjusted and controlled by differences in age, sex, comorbidities (malignancy and neutropenic fever), SAPS 3, SOFA score, and septic shock or sepsis. We first proceeded with identifying some unusual observations that the values of biomarker clearances were extremely low. In the interest of finding a common model for usual observations, we set aside these 9 episodes. Further explanation is given in the Discussion section. A stepwise regression selection results are then used to identify possible risk indicators and summarized in Table 4.

**Table 4:** Multiple logistic analysis for biochemical markers in predicting treatment failure and 28-day mortality.

	Treatment failure		28-day mortality	
	Adjusted OR (95% CI)	p	Adjusted OR (95% CI)	p
PCTc Peak-min	0.980 (0.962–0.998)	0.027	0.986 (0.971–1.001)	0.064
CRPc Peak-min	0.982 (0.966–0.998)	0.030	0.984 (0.968–1.001)	0.062
SAPS 3	-*	-	1.061 (1.025–1.099)	< 0.001
SOFA score	1.146 (1.018–1.292)	0.025	-*	-

PCTc Peak-min, procalcitonin clearance; CRPc Peak-min, C-reactive protein clearance; SAPS 3, simplified acute physiology score 3; SOFA, sequential organ failure assessment score;

* the variables did not retain statistical significance (*p*-value > 0.2) and were not kept in respective outcome analyses.

**Fig 1:**
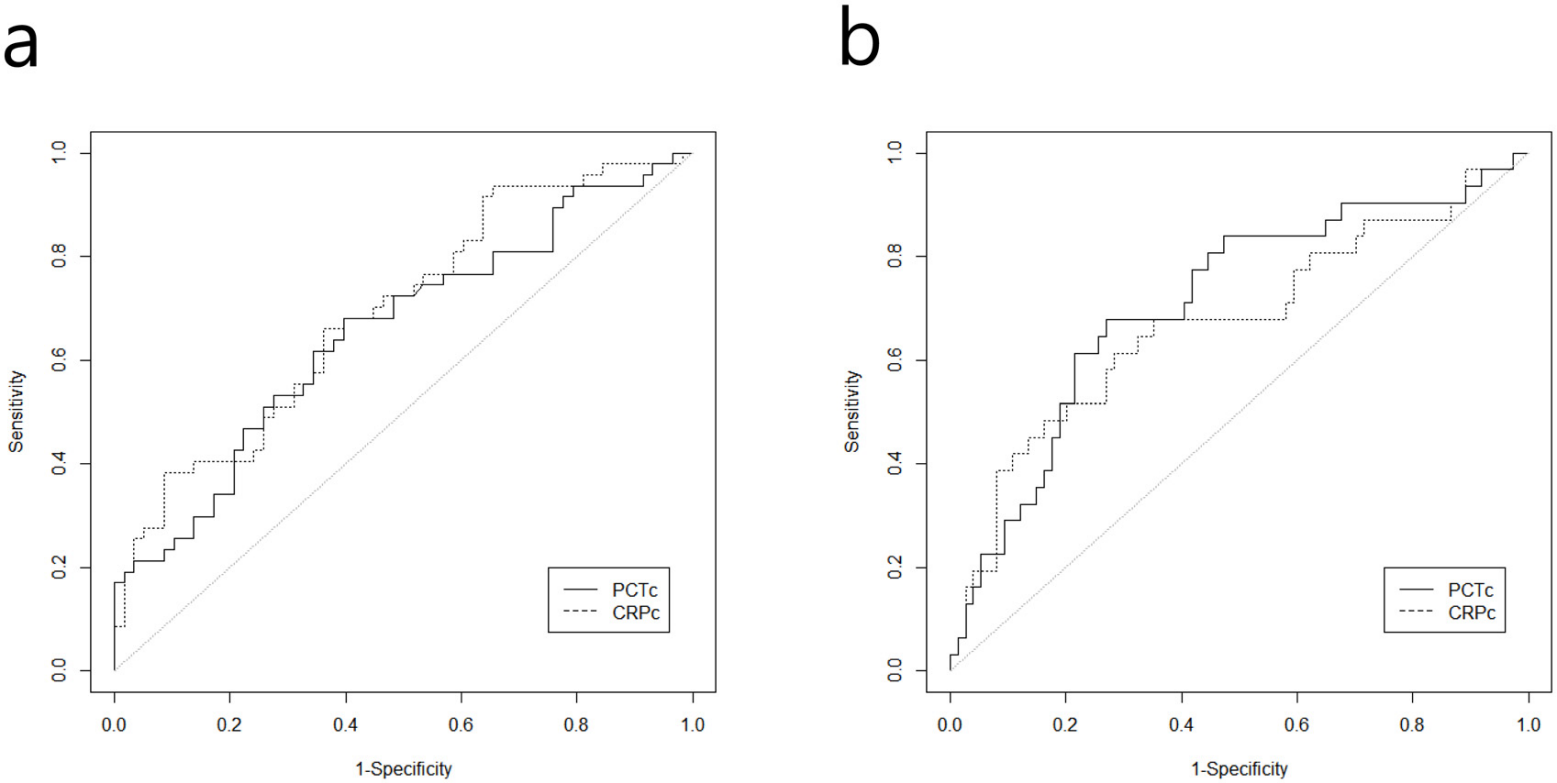
(a) Receiver operating characteristic (ROC) curves for procalcitonin (PCTc) and C-reactive protein (CRPc) clearance predict treatment failure. (b) Receiver operating characteristic (ROC) curves for procalcitonin (PCTc) and C-reactive protein (CRPc) clearance predict 28-day mortality. The test for AUC difference between the two markers had a p-value > 0.2 for both treatment failure and 28-day mortality.

It appears that both are independently significant markers in predicting treatment failure and complementing each other. To determine which marker has a better predictability, we compared models with PCTc and CRPc separately to the one with both of them in the model. We find that both single marker models’ predictable power is similar in terms of adjusted AUCs; they were 0.71 (0.61–0.82) for PCTc and 0.71 (0.61–0.81) for CRPc for predicting treatment success. When both markers are used, the adjusted AUC rises to 0.74 (0.64–0.84) for predicting treatment success. When we applied these two markers to predict the 28 day mortality, they were significant marginally (0.05 < *p*-value < 0.10). However, we retain them in the model from the clinical consideration. Similarly, the adjusted AUCs for predicting 28 day mortality were 0.77 (0.66–0.88) and 0.77 (0.67–0.88) for PCTc and CRPc, respectively. However, there were no differences between the AUCs of PCTc and CRPc among treatment failure (*p* = 0.531) and 28-day mortality, (*p* = 0.553) (Fig 1). When both markers are used, the adjusted AUC rises to 0.79 (0.69–0.90) for 28 day mortality. However, when we factor others into the consideration such as the cost of these markers, it is evident that CRPc is actually superior to PCTc.

**Fig 2:**
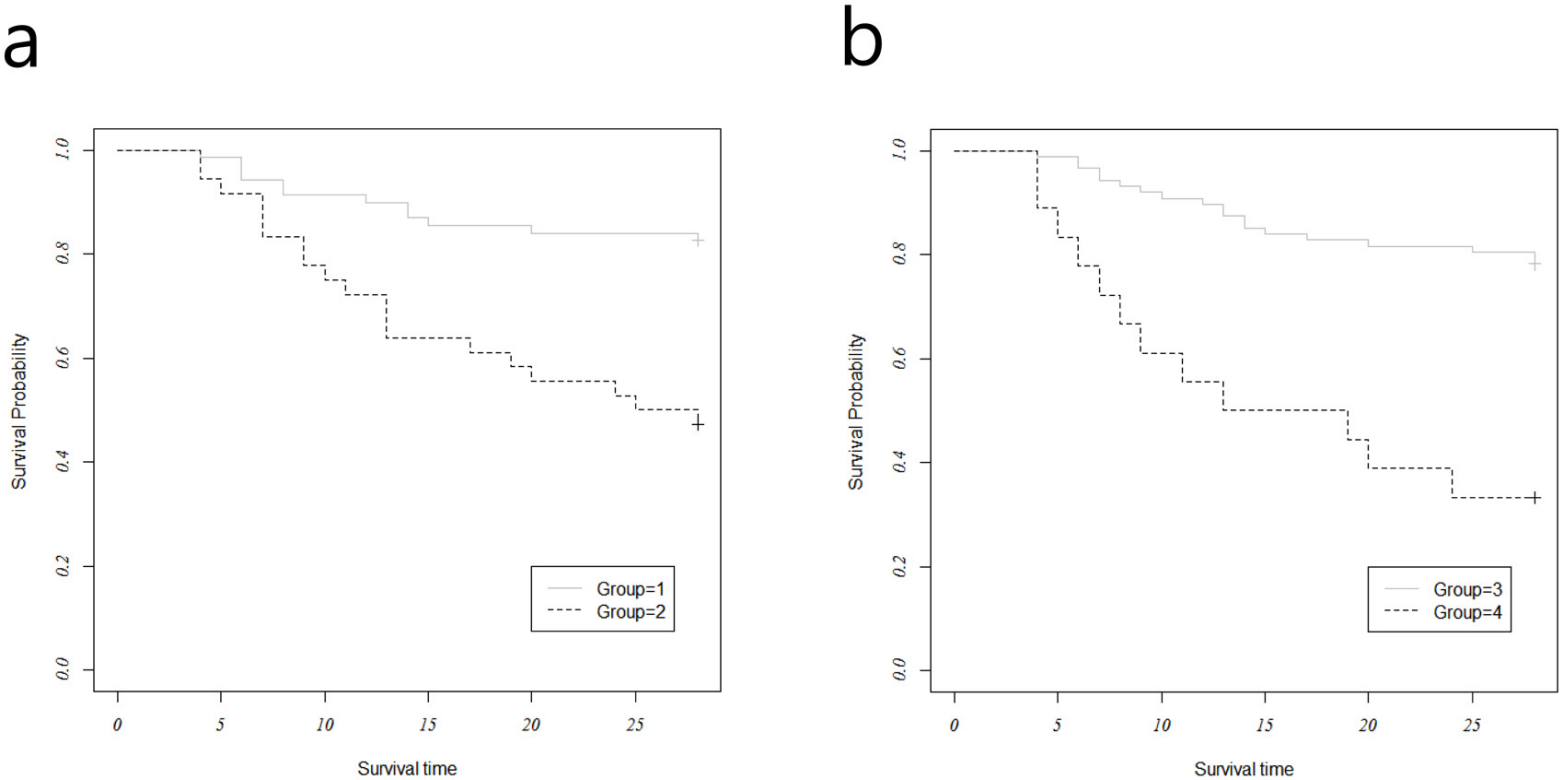
(a) Kaplan-Meier 28-day survival analyses comparing groups 1 (PCTc ≥ 78%) and 2 (PCTc < 78%). Solid line, group 1; dotted line, group 2; *p* = 0.001 based on log-rank tests. (b) Kaplan-Meier 28-day survival analyses comparing groups 3 (CRPc ≥ 36%) and 4 (PCTc < 36%). Solid line, group 3; dotted line, group 4; *p* < 0.001 based on log-rank tests.

Mortality rates were lower in groups with higher clearances of PCT or CRP (Fig 2). A cut-off of 78% was used to stratify patients into those with PCTc ≥ 78% (Group 1) and those with PCTc < 78% (Group 2). The rates of 28-day mortality were significantly lower among those with high levels of PCTc, compared to those with low levels of PCTc (17% vs. 53%, log-rank test, *p* = 0.001). A cut-off of 36% was used to stratify patients into those with CRPc ≥ 36% (Group 3) and those with PCTc < 36% (Group 4). The rates of 28-day mortality were significantly lower among those with high levels of CRPc, compared to those with low levels of CRPc (22% vs. 67%, Log-rank test, *p* < 0.001)

## Discussion

Our study found changes in PCT and CRP concentrations were associated with patient outcome, including treatment response and survival. Absolute PCT and CRP levels were not associated with outcome. Clearance of PCT and CRP showed better survival rates in 28-day survival curves. Although PCTc and CRPc were associated with outcomes, PCTc was not superior to CRPc for predicting treatment response and mortality in our study. We noticed that some values of biomarker clearances were extremely low. Extremely abnormal biomarker clearances were resulted when initial baseline levels of biomarkers were nearly normal and subsequent levels were extremely high. It was possible when we obtained blood samples on a very early stage of sepsis or concentrations of biomarkers were changed very slowly.

CRP is a traditional marker of sepsis. In 1930, Tillet and Francis identified the capacity to precipitate polysaccharide fractions, designated fraction C, from Streptococcus pneumonia in sera from patients with pneumonia [25]. CRP is the most frequently used biomarker in clinical practice. Previous studies have shown daily CRP measurements are useful for monitoring the course of sepsis in critically ill patients, and may be used to indicate successful treatment [25, 26]. CRP monitoring represents a possible means of stopping antibiotics safely, sparing patients from drug toxicity likely decreasing the risk of resistance and decreasing costs. Normalization of CRP concentrations has been proposed as a guideline for stopping antibiotics. In addition, CRP is an inexpensive, consistent, and reproducible test that is available in most hospitals [25].

Previous studies have shown biochemical markers help diagnose sepsis and are associated with patient outcome in severe sepsis and septic shock [1, 3, 5, 27]. Although the diagnostic accuracy of PCT was higher than CRP in sepsis [1–5], it was unclear which biomarker had more prognostic accuracy in critically ill septic patients. Several recent studies found CRP has higher prognostic value than PCT and that both biochemical markers have similar predictive value for determining the outcome of septic patients. Hoeboer et al. reported that CRP might be favored over PCT courses in judging responses to antibiotic treatment. PCT, however, may better indicate the risk of complications, including bloodstream infection, septic shock, organ failure, and mortality [6]. Seligman et al. found that changes of PCT and CRP at onset and on the fourth day can predict survival of ventilator-associated pneumonia patients. A decrease in either one of these marker values predicts survival [9]. Park et al. reported that CRP is more accurate than PCT for predicting infection in patients with impaired renal function [28]. A recent study found that median CRP concentrations were higher in non-survivors than survivors (105 mg/L vs. 44 mg/L) after the 3^rd^ to 5^th^ day of treatment. Rather than initial CRP concentrations, CRP concentrations measured a few days after admission may be more helpful for physicians to evaluate treatment response and sepsis outcome in the ICU. CRP levels greater than 100 mg/L on the third day in the ICU may be as useful of a predictor of mortality as high SOFA scores [29]. Oliveira et al. determined that CRP was as useful as PCT in reducing antibiotic use in septic patients, causing no apparent harm. In their study, a PCT-based protocol was not superior to a protocol based on serum CRP levels for reducing antibiotic use. Remarkably, the length of antibiotic therapy was shorter in the CRP group and less than the maximum therapy duration proposed [28]. Recently, several studies found that CRP is as beneficial as PCT in predicting outcomes and reducing antibiotic use in septic patients [6, 9, 28, 29]. In addition, CRP is more cost effective than PCT. PCT testing is eight times more expensive than CRP testing in Korea, and two to four times more expensive in the United States and Europe [28]. Although beneficial effects of CRP have been reported, other studies showed initial PCT concentrations or changes in PCT concentration have higher prognostic value than CRP in sepsis [1, 2, 4, 27, 30].

Hoeboer et al. designed their study differently compared to other studies. They did not use initial concentrations of biochemical markers as baseline concentrations. The initial baseline of infectious markers was defined as peak levels within 2 days [6]. Robinson at al. showed PCT peak occurred on day 2 in febrile neutropenia after fever onset. A higher level of PCT peak occurred after 3 days of persistent fever in invasive fungal diseases [31]. Initial PCT concentrations may not reflect the infection severity in febrile neutropenia and fungal infection because PCT concentrations have a tendency to exhibit delayed peak levels. In our study, there was a high proportion of patients with febrile neutropenia and immunocompromised states. CRP can also slowly change in septic patients [6]. Thus, we chose peak levels of these biochemical markers within two days as the initial baseline because early peak levels of PCT or CRP may approximately represent clinical deterioration onset and sepsis severity. We defined subsequent levels of biomarkers using a similar method as baseline. This method might be helpful for analyzing the association of changes in these markers and patient prognosis in this study.

This study has several limitations. This is retrospective review of medical records and was performed in single center. Moreover, the sample population was not large. Time zero was difficult to define because of the retrospective nature of study. The institutional rapid response team was activated at the emergency room or general ward when the septic patient was critically ill at our hospital. Therefore, most septic patients were admitted to the ICU within 24 hours and there were no large gaps in clinical deterioration and ICU admission. There was a high proportion of patients with malignancies and febrile neutropenia in this study. Furthermore, the underlying diseases were heterogeneous. The proportion of patients with malignancies in relapsed/refractory states and or with extensive disease was also high. A significant numbers of patients had high SAPS 3 and SOFA scores. These factors could affect the prognosis of patients. Although we found not all of these factors to be significant in our model, it may be due to a small sample size. Nevertheless, the predictable power of PCPc and CRPc remains uninfluenced by the small sample size and the treatment success and 28 day mortality are successfully indicated. Further research may need to confirm our findings.

## Conclusions

Changes in PCT and CRP concentrations were associated with the outcomes of critically ill septic patients. Changes in CRP concentrations were not inferior to changes in PCT concentrations statistically for predicting treatment response and survival. While further research is needed to confirm our findings, we propose that CRP may be as effective as PCT in predicting the outcome of critically ill patients with severe sepsis and septic shock. In addition, CRP testing is more cost effective and readily available than PCT testing, which makes CRP testing superior to PCT.
